# Study of Functional Outcome and Postoperative Complications Among Proximal Humerus Fracture Patients Treated With Proximal Humerus Internal Locking System (PHILOS) Plating

**DOI:** 10.7759/cureus.42411

**Published:** 2023-07-24

**Authors:** Rohit Jhamnani, Manjeet Singh Dhanda, Avinash Surana

**Affiliations:** 1 Orthopaedics, Pacific Medical College and Hospital, Udaipur, IND; 2 Orthopaedics, Shaheed Hassan Khan Mewati (SHKM) Government Medical College, Nuh, IND; 3 Preventive Medicine, Infantry Division, 14 Coprs, Bareilly, IND

**Keywords:** postoperative, internal locking, elderly individuals, proximal humerus, fracture

## Abstract

Background

Proximal humerus fractures comprise nearly 4%-5% of all fracture types and nearly 25% of fracture humerus. These fractures are commonly seen in the elderly population (people aged 60 years or more). The best way to treat elderly people who have three- or four-part fractures of the proximal humerus is debatable, with many in favour of prosthetic humeral head replacements. This study aimed to assess the functional outcome of proximal humerus fractures managed with a proximal humerus locking plate and to investigate the incidence of complications in these patients.

Methodology

This retrospective study included 32 cases of proximal humerus fractures managed surgically at a tertiary care teaching hospital in Rajasthan between July 2016 to July 2022 with a proximal humerus internal locking system (PHILOS) plate. NEER scoring system was used to categorise the fractures. Functional assessment was done using Constant Murley's shoulder score. Constant Murley shoulder score was graded as failure (0-69 points), unsatisfactory (70-79), satisfactory (80-89), and excellent (90-100). Subjects having metastatic and pathological fractures; associated fractures in the ipsilateral limb; having major nerve injury and cases of open fracture were excluded from this study.

Results

The mean age was 54.5±6.4 years. Of the total 32 cases of proximal humerus fractures, 12 cases of two-part fractures received an excellent grade, compared to seven cases of three-part fractures. Three two-part fracture cases and two three-part fracture cases all received satisfactory grades. Excellent results (score > 89) were noticed in 62.5% (n = 20) of the instances, satisfactory results (scoring 80-89) in 21.87% (n=7), poor results (score 70-79) in 9.38% (n=3), and failure results (score 70) in 6.25% (n=2). In 65.6% of cases, follow-up showed no complications. Shoulder stiffness and malunion (9.38%) were the most frequent consequences, followed by avascular necrosis (6.25%).

Conclusions

Based on the findings of this retrospective study, it can be opined that PHILOS plaiting appears to be a secure option for proximal humerus fracture cases. It offers solid fixation, prompt mobilisation, and excellent functional outcomes as observed in this study. Additionally, very few post-operative complication rates again support our conclusion.

## Introduction

Proximal humerus fractures comprise nearly 4%-5% of all fracture types and nearly 25% of fracture humerus. These fractures are commonly seen in the elderly population (people aged 60 years or more). The proximal humerus typically breaks into four fragments along the physeal lines of fusion - two tuberosities, the humeral head, and the shaft. Most tuberosity fractures take place secondary to the displacement of the head fragment and their degree of spatial displacement is initially minimal, relative to their normal anatomic position [1]. With non-operative management, progressive displacement may occur because of the unopposed pull of the rotator cuff muscles. Hence, the non-operative management of these displaced fractures is more controversial. Non-operative treatment may result in complications like non-union, osteonecrosis, and malunion. Hence, in the majority of cases, operative management becomes mandatory for better outcomes [2].

Over the past 10 years, there has been considerable expansion in the range of reconstructive implants available to treat these injuries. There are different methods of internal fixation using, locking compression plates and screws, percutaneous fixation with metallic k wires and screws, tension band, external fixation, fixed-angle blade plates, transosseous suture fixation, intramedullary device shoulder arthroplasty, but none of these methods have been successful [3]. The management of these fractures can be a significant challenge, especially in the presence of poor cancellous bone due to osteoporosis and multiple fracture segments resulting in failure of fixation with conventional plating systems. The major goal in the treatment of this fracture is to promote complication-free healing to recreate a pain-free mobile, stable and functional shoulder joint [4,5].

The proximal humerus internal locking system (PHILOS) is an anatomical locking plate that was created by the AO/OTA (American orthopaedic foundation and orthopaedic trauma association) to improve functional outcomes, particularly for osteoporotic patients [6]. PHILOS enables angled stabilization with multiple interlocking screws than conventional plates. The ability of screws to lock provides better anchorage in the osteoporotic bone to a plate gives angular stability to the construct and maintains postoperative reduction during early functional rehabilitation and avoids joint stiffness and enhances functional outcome [7]. This study aimed to assess the functional outcome of proximal humerus fractures managed with open reduction and internal fixation with a PHILOS plate and to investigate the incidence of complications in the patients.

## Materials and methods

In this retrospective study, 32 cases of proximal humerus fractures treated surgically with a PHILOS plate at a tertiary care teaching hospital in Rajasthan between July 2016 and July 2022 were included. The Institutional Ethics Committee (IEC) approval was taken before the initiation of the study (approval letter no. IEC/PMC/22/76).

Inclusion criteria and exclusion criteria

All the adult cases presenting with displaced proximal humerus fracture managed operatively with a proximal humerus locking plate were included in the study. Subjects with metastatic and pathological fractures; associated fractures in the ipsilateral limb; having major nerve injury (e.g., axillary nerve or deltoid palsy), and cases of open fracture were excluded.

This study used the NEER scoring system to categorise the fractures as it is a standardised shoulder score. NEER score was given by the American Academy of Orthopaedic Surgeons [8]. The four evaluation criteria for this scoring system are Pain, Function, Range of motion, and Anatomy. The scoring cap is 100.

Demographic information, the date of the injury, the date of the operation, the interval between the two, the surgical approach and fixation, and the wound problems before and after the operation were all noted. Patients were checked for local infection, range of motion, and pain during the follow-up visits. All patients were followed up at six weeks, 12 weeks, and at six months. A functional assessment was also performed. Additionally noticed were alignment, fracture union, implant loosening, loss of reduction, and avascular necrosis signs.

Functional assessment was done using Constant Murley's shoulder score. Constant Murley shoulder score was graded as failure (0-69 points), unsatisfactory (70-79), satisfactory (80-89), and excellent (90-100). Radiographs were taken at each follow-up to evaluate fracture union and any complication like fracture displacement, loss of reduction or varus, or valgus angulation was also noted. Failure was defined as backing out of the screw, plate breakage/pull-out, malunion, nonunion or avascular necrosis of the humeral head.

Statistical analysis

Descriptive statistics of the sociodemographic profile of the subjects in the form of frequencies and percentages were calculated and presented. For quantitative variables, mean and standard deviation (SD) were reported as measures of central tendency and dispersion, respectively. Data analysis was performed using a statistical software namely SPSS Statistics version 27.0.1.0 (IBM Corp., Armonk, NY, USA).

## Results

This study included data from all 32 cases of proximal humerus fractures treated surgically with the PHILOS. The mean age was 54.5±6.4 years with approximately half of the patients in the age group of 41-59 years (53.1%, n=17). Gender wise males (78.1%, n=25) outnumbered female cases. The mean Constant Murley shoulder score was 85.8. 

According to NEER’s classification of fractures, 46.88% (n=15) cases had two-part fractures, 37.49% (n=12) cases had three-part fractures and 9.38% (n=3) cases had four-part fractures whereas 6.25% (n=2) cases had fracture-dislocation. Of the total 32 cases, 18 (56.25%) subjects had a history of road traffic accidents whereas 14 (43.75%) cases had a history of falls (Table 1).

**Table 1:** Distribution of subjects as per the mode of injury and type of fracture

Type of fracture as per NEER classification	Prevalence of injury	Mode of injury	
		RTA	Fall
	No. of subjects (%)	No. of subjects (%)	No. of subjects (%)
2 Part (NEER type 2)	15 (46.88%)	8 (25%)	7 (21.8%)
3 Part (NEER type 3)	12 (37.49%)	7 (21.8%)	5 (15.6%)
4 Part (NEER type 4)	3 (9.38%)	2 (6.25%)	1 (3.12%)
Fracture dislocation	2 (6.25%)	1 (3.12%)	1 (3.12%)
Total	32 (100%)	18 (56.25%)	14 (43.75%)

The excellent grade was noted in 12 cases of two-part fracture and in seven cases of three-part fracture. A satisfactory grade was observed in three cases of two-part fracture and in two cases of three-part fracture. Failure grade was observed in one case each of three-part fracture and fracture-dislocation (Table 2).

**Table 2:** Distribution of subjects as per functional outcome against the type of fracture (n=32)

Grading of functional outcome	Type of fracture			
	Two-part (n=15)	Three-part (n=12)	Four-part (n=3)	Fracture dislocation (n=2)
Excellent	12	7	1	-
Satisfactory	3	2	1	1
Unsatisfactory	-	2	1	-
Failure	-	1	-	1

The outcome was noted as excellent (score >89) in 62.5% (n=20) cases, unsatisfactory (score 70-79) in 9.38% (n=3) cases and failure (score <70) in 6.25% (n=2) cases (Table 3).

**Table 3:** Distribution of subjects as per functional outcome and NEER Score (n=32)

Grading of functional outcome	NEER Score	No. of subjects	Percentage
Excellent	>89	20	62.5
Satisfactory	80-89	7	21.87
Unsatisfactory	70-79	3	9.38
Failure	<70	2	6.25

Amongst 32 operated cases, follow-up revealed no complication in 65.6% of cases (n=21). The most common complication seen was stiffness of the shoulder and malunion in 9.38% (n=3) subjects each followed by avascular necrosis seen in 6.25% (n=2) subjects (Table 4). Stiffness of the shoulder improved with physiotherapy.

**Table 4:** Distribution of subjects as per post-operative complications (n=32)

Post-operative complication	No. of subjects	Percentage
No complication	21	65.6
Stiffness of shoulder	3	9.38
Malunion	3	9.38
Avascular necrosis	2	6.25
Penetration of screw into joint space	1	3.12
Loosening of implant	1	3.12
High placement of the plate	1	3.12

## Discussion

In this study, the mean age was 54.5±6.4 years, which was similar to the age incidence observed by Egol et al. [9]. About half of the patients were in the age range of 41 to 59 years. Out of a total of 32 cases, 14 (43.75%) individuals had a history of a fall, compared to 18 (56.25%) subjects who had a history of RTA. These results are consistent with previous research by Kirsch et al. who found that out of 40 cases evaluated, 47.5% included traffic accidents, 50% had a history of falling, and 2.5% had a history of assault [10].

According to NEER's classification of fractures, 6.25% of cases in our study showed fracture dislocation, whereas 46.88% of cases had two-part fractures, 37.49% had three-part fractures, and 9.38% had four-part fractures. The mean constant Murley score was observed to be 85.8 at the end of the follow-up period in this study. In another research by Yadav et al., in which 44 patients with fresh three- and four-part fractures of the proximal humerus were treated surgically with open reduction and internal fixation using the PHILOS system in 21 patients and closed reduction and internal fixation with k-wire in 23 patients. The mean constant Murley score in patients treated with closed reduction and internal fixation with k-wire was 79.48 whereas patients treated with open reduction and internal fixation using PHILOS had a mean constant Murley score of 85.29 at the end of the follow-up period [11].

The mean constant Murley shoulder score, according to a study by Jagiasi et al., was 61.8. The mean constant score was 50.53 for people over 45 years of age and 72.91 for people under 45 years of age [12]. A study from Madhya Pradesh noticed that the mean constant score in the delto splitting approach was 70.9 and 74 in a deltopectoral group in a study among 26 cases. All fractures united radiologically and clinically and the constant Murley score at the final follow-up was 72.5. At the final follow-up, eight patients had good scores, 10 patients had moderate scores, six patients had excellent outcomes and two patients had poor outcomes according to constant Murley score [13].

A different study from Gujarat found that excellent results occurred in 54% of instances, satisfactory results in 24% of cases, unsatisfactory results in 12% of cases, and failures occurred in 10% of cases [14]. Ganesan et al. also observed excellent results in 50% of the instances, satisfactory results in 30% of the cases, unsatisfactory results in 10% of the cases, and failure results in 10% of the cases [15]. According to a study by Jagiasi et al., the results were outstanding in 40% of the instances, very good in 6.66%, good in 30%, fair in 20%, and poor in 3.33% of the cases [12]. The results of a study by Vijayanand et al. were excellent outcomes in 23 cases, satisfactory in four, unsatisfactory in two, and in one case a failure [16]. According to a study by Bansal et al., the results were excellent in 16% of the instances, good in 44%, fair in 16%, and poor in 24% of the patients [17].

In this study, the most frequent complications were shoulder stiffness and malunion, both occurring in 9.38% (n=3) of the subjects, and avascular necrosis, which was observed in 6.25% (n=2) of the subjects. These outcomes are comparable to those of a study conducted by Pandya et al. in Gujarat. In 9.75% of cases, the authors noted shoulder discomfort and malunion [14].

Thanasas et al. [18] reported an impingement rate of 5.5% in a comprehensive review of 12 studies on proximal humerus fractures. Seven per cent (3/41) of respondents reported stiffness. All of these patients were over 65, had diabetes, and were discovered to have neglected to perform the recommended postoperative physical therapy exercises. Only one patient (2%) out of the total had a surface infection that needed to be treated with intravenous antibiotics. Brunner et al. [19] and Agudelo et al. [20] observed infection rates of 2% and 4.5%, respectively, in their trials. One patient who underwent locking proximal humeral plate (LPHP) treatment for a two-part fracture had a varus malunion (2%) but was able to carry out her daily activities with a moderate functional result; therefore, the revision was not considered.

Limitations

This study attempted to address an important debatable issue of finding the best way to treat elderly patients who have three- or four-part fractures of the proximal humerus. First and foremost, study design (a retrospective study) is an evident limitation. Secondly, this study was conducted on a relatively small number of subjects and restricted to only one centre. Thirdly, no comparison group was taken in this study. Results could be compared between two groups like open reduction and internal fixation using PHILOS versus closed reduction and internal fixation with k-wire.

## Conclusions

Based on the findings of this retrospective study, it can be opined that PHILOS plaiting appears to be a secure option for proximal humerus fracture cases. It offers solid fixation, prompt mobilisation, and excellent functional outcomes as observed in this study. Additionally, very few post-operative complication rates again support our conclusion.
